# Supplementary material for: High-throughput targeted long-read single cell sequencing reveals the clonal and transcriptional landscape of lymphocytes
Source: Nat Commun. 2019 Jul 16;10:3120. doi: 10.1038/s41467-019-11049-4 (PMC6635368; doi:10.1038/s41467-019-11049-4)
Supplement: Supplementary file 1 — Supplementary Information [file 41467_2019_11049_MOESM1_ESM.pdf]

High-throughput targeted long-read single cell sequencing reveals the clonal and transcriptional landscape of lymphocytes

Singh et al.

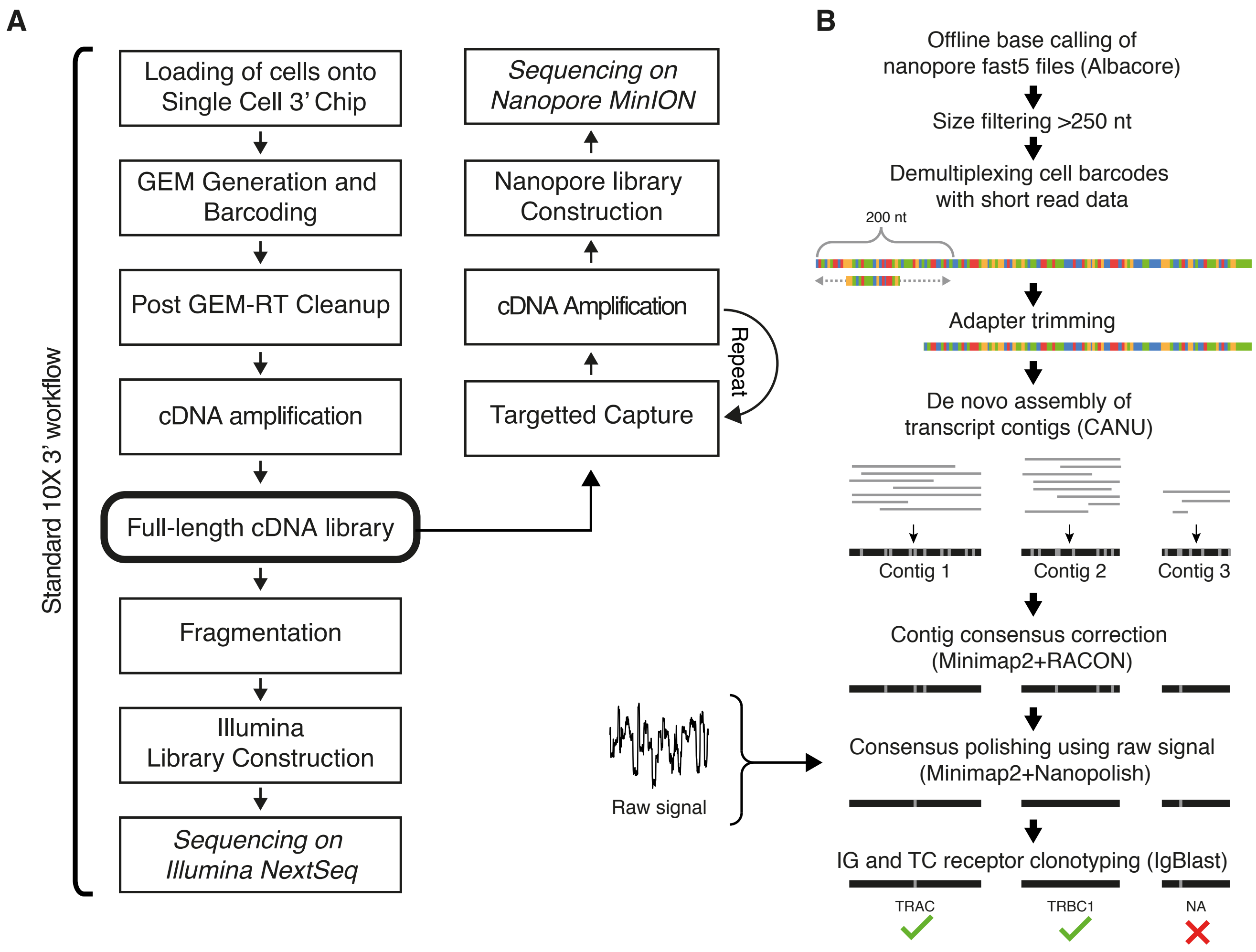

Supplementary Figure 1. RAGE-Seq experimental protocol and computational pipeline. **(a)** Detailed experimental workflow of RAGE-Seq. Single-cell suspensions are loaded onto 3' library chips for the Chromium Single Cell 3' Library according to the manufacturer's recommendations (10X Genomics). Single cells are partitioned into Gel Beads in Emulsion (GEMs) with cell lysis and barcoded reverse transcription of RNA. Following isolation of GEMs from the Chromium instrument, full-length cDNA is PCR amplified. At this point the cDNA library is split into two fractions. The first fraction undergoes the remainder of the Chromium workflow which involves shearing, adapter ligation and Illumina sequencing. The second fraction undergoes two rounds of targeted capture of TCR and BCR genes followed by ONT library preparation using the 1D adapter ligation sequencing kit and sequencing on the MinION platform. **(b)** Detailed computational pipeline of RAGE-Seq.

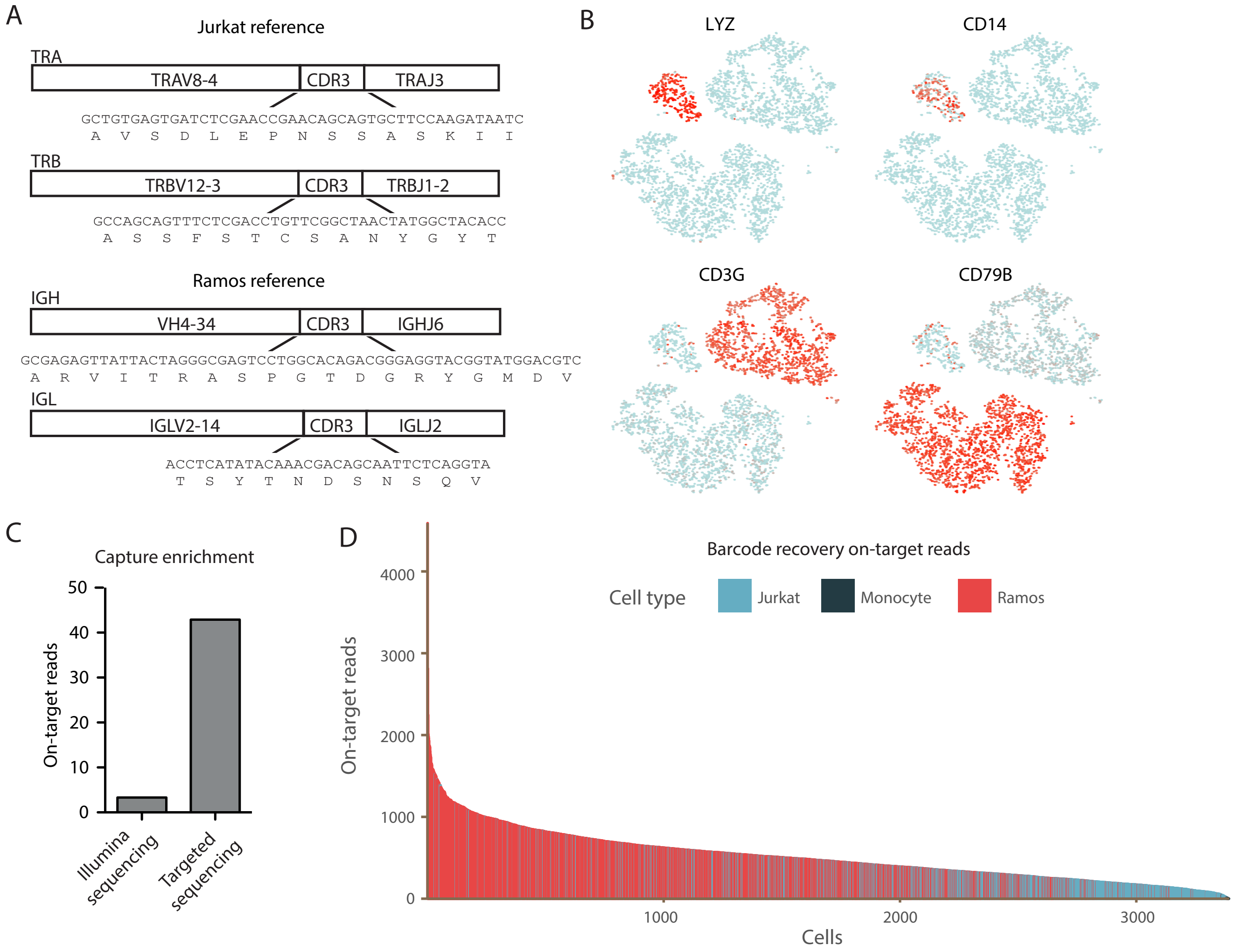

Supplementary Figure 2. RAGE-Seq cross-sequencing platform quality control measurements. **(a)** Reference CDR3, V and J gene segments that encode the TCRα and TCRβ chains of Jurkat or the immunoglobulin heavy and light chains of Ramos. For Ramos the most abundant heavy chain CDR3 (1123/1278 cells) and light chain CDR3 sequences (821/937) were chosen as the reference. **(b)** tSNE analysis of key canonical gene expression markers used to identify the cell type of each cluster. *CD3G* was used to identify Jurkat, *CD79B* was used to identify Ramos and *LYZ* and *CD14* were used to identify monocytes. **(c)** The relative enrichment of targeted capture of antigen receptor genes. On-target reads were determined by the percentage of total Nanopore or Illumina sequencing reads that align to *TRA*, *TRB*, *IGH*, *IGL* and *IGK* constant region genes. **(d)** Nanopore cell barcode recovery for Nanopore reads that are on-target. Mean on-target reads per cell type: Jurkat, 309; Ramos, 646; Monocyte, 1.49. Number of barcodes recovered: Jurkat, 1454/1463; Ramos, 2000/2000; Monocyte, 130/280.

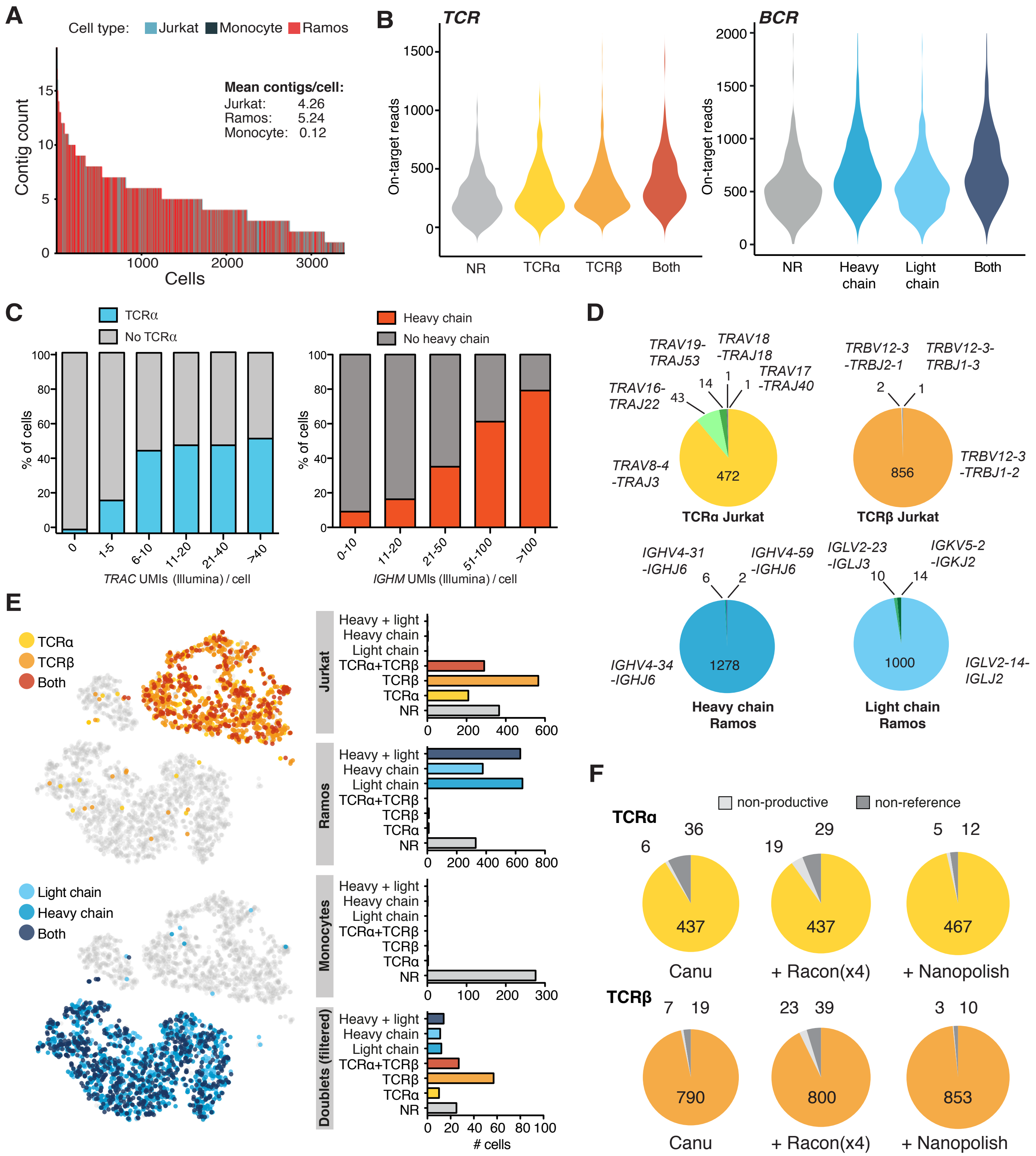
A
Cell type: Jurkat Monocyte Ramos
Mean contigs/cell:
Jurkat: 4.26
Ramos: 5.24
Monocyte: 0.12
Contig count
Cells
B
TCR
BCR
On-target reads
NR
TCRα
TCRβ
Both
Heavy chain
Light chain
C
TCRα
No TCRα
Heavy chain
No heavy chain
% of cells
TRAC UMIs (Illumina) / cell
IGHM UMIs (Illumina) / cell
D
TRAV19-TRAJ53
TRAV18-TRAJ18
TRAV17-TRAJ40
TRAV16-TRAJ22
TRAV8-4-TRAJ3
TCRα Jurkat
TRBV12-3-TRBJ2-1
TRBV12-3-TRBJ1-3
TRBV12-3-TRBJ1-2
TCRβ Jurkat
IGHV4-31-IGHJ6
IGHV4-59-IGHJ6
IGHV4-34-IGHJ6
Heavy chain Ramos
IGLV2-23-IGLJ3
IGKV5-2-IGKJ2
IGLV2-14-IGLJ2
Light chain Ramos
E
TCRα
TCRβ
Both
Light chain
Heavy chain
Both
Jurkat
Ramos
Monocytes
Doublets (filtered)
Heavy + light
Heavy chain
Light chain
TCRα+TCRβ
TCRβ
TCRα
NR
cells
F
non-productive
non-reference
TCRα
TCRβ
Canu
+ Racon(x4)
+ Nanopolish

Supplementary Figure 3. Quality control measurements of antigen receptor assembly. **(a)** Mean number of contigs assembled per cell following *de novo* assembly and polishing of nanopore reads. Each bar corresponds to an individual cell. Mean number of contigs per cell for each cell type: Jurkat, 4.26; Ramos, 5.24; Monocyte, 0.12. **(b)** The number of on-target nanopore reads for Jurkat (left panel) or Ramos (right panel) grouped by the recovery of TCR chains or BCR chains, respectively. NR, no receptor. Only productive TCR and BCR chains that match their reference V and J gene were assigned. **(c)** The recovery of Jurkat cells assigned a TCRα chain (left panel) or Ramos cells assigned a immunoglobulin heavy chain (right panel) as a function of the number of Illumina *TRAC* (Jurkat) or *IGHM* (Ramos) UMIs per cell. **(d)** Assignment of TCR chains to Jurkat cells or BCR chains to Ramos cells based on their V and J gene segment usage. Shown in each pie graph is the number of cells expressing the designated V and J genes. **(e)** t-SNE plot of Jurkat, Ramos and monocyte cells assigned TCR (top panel) or BCR (bottom panel) chains. Right panels show the total number of cells assigned different chains for each cell type. Doublets (n=136 cells) are not shown on the t-SNE plots and were filtered out based on high gene count (see Methods). **(f)** Accuracy of CDR3 sequences of Jurkat cells at each stage of contig assembly and polishing. Shown are the number of cells assigned a CDR3 sequence that match the reference *TRA* or *TRB* CDR3. 'Non-reference' refers to a CDR3 sequence that does not match the reference Jurkat CDR3. 'Non-productive' refers to TCR chains with a CDR3 sequence that is out-of-frame or contains stop-codons and are usually filtered from the dataset. Only TCR chains that match the Jurkat reference V and J gene segments are assigned.

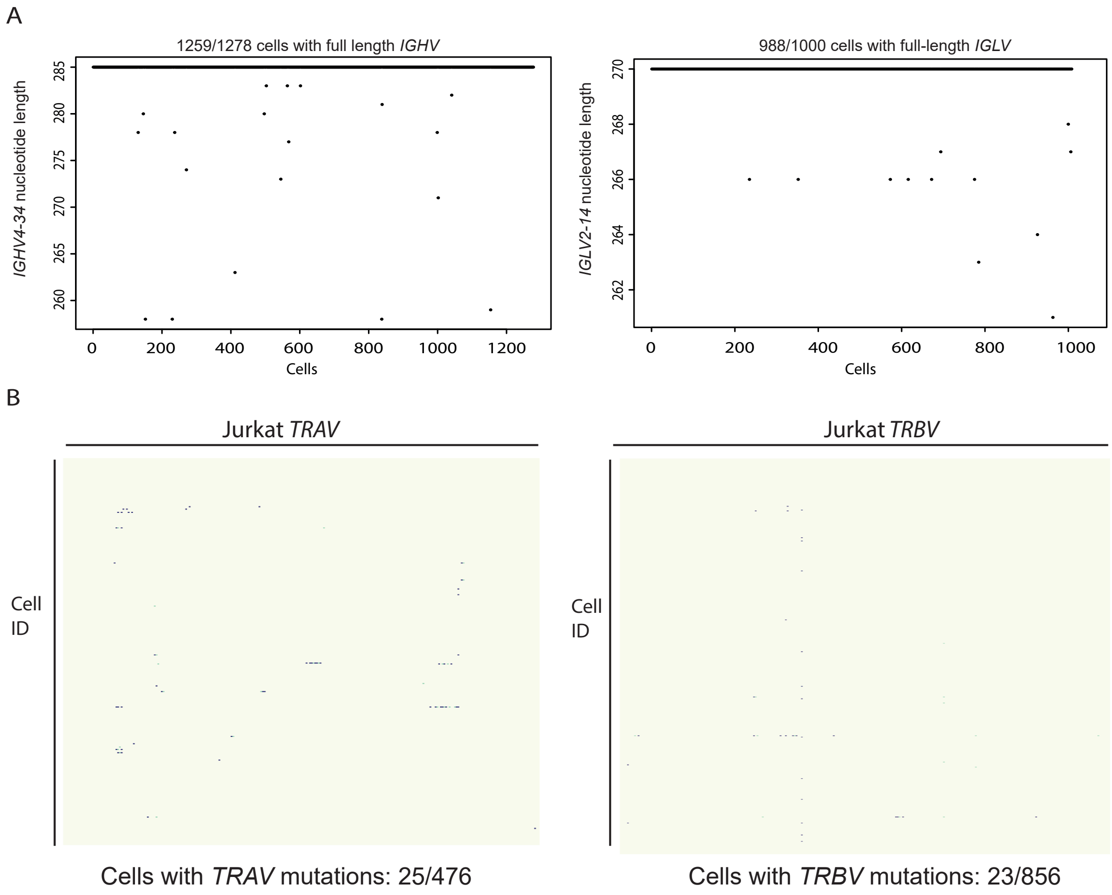

Supplementary Figure 4. Quality control measurements of calling somatic hypermutation in individual cells. **(a)** Nucleotide length of Ramos heavy chain (left panel) and light chain (right panel) V regions for each individual Ramos cell. The number of cells with the maximum length of the entire *IGVH4-34* (left) or *IGLV2-14* (right) gene is indicated. **(b)** Heatmap of the V regions of individual Jurkat cells encoding the TCRα (left panel, n=476) and TCRβ (right panel, n=856) chain. Each row represents an individual cell and each column a nucleotide position in the respective V gene. Light blue represents synonymous nucleotide substitutions while dark blue represents non-synonymous nucleotide substitutions, when compared to germline *TRAV8-4* and *TRBV12-3* sequences. Length of Jurkat *TRAV8-4*: 274 nt; length of Jurkat *TRBV12-3*: 276 nt.

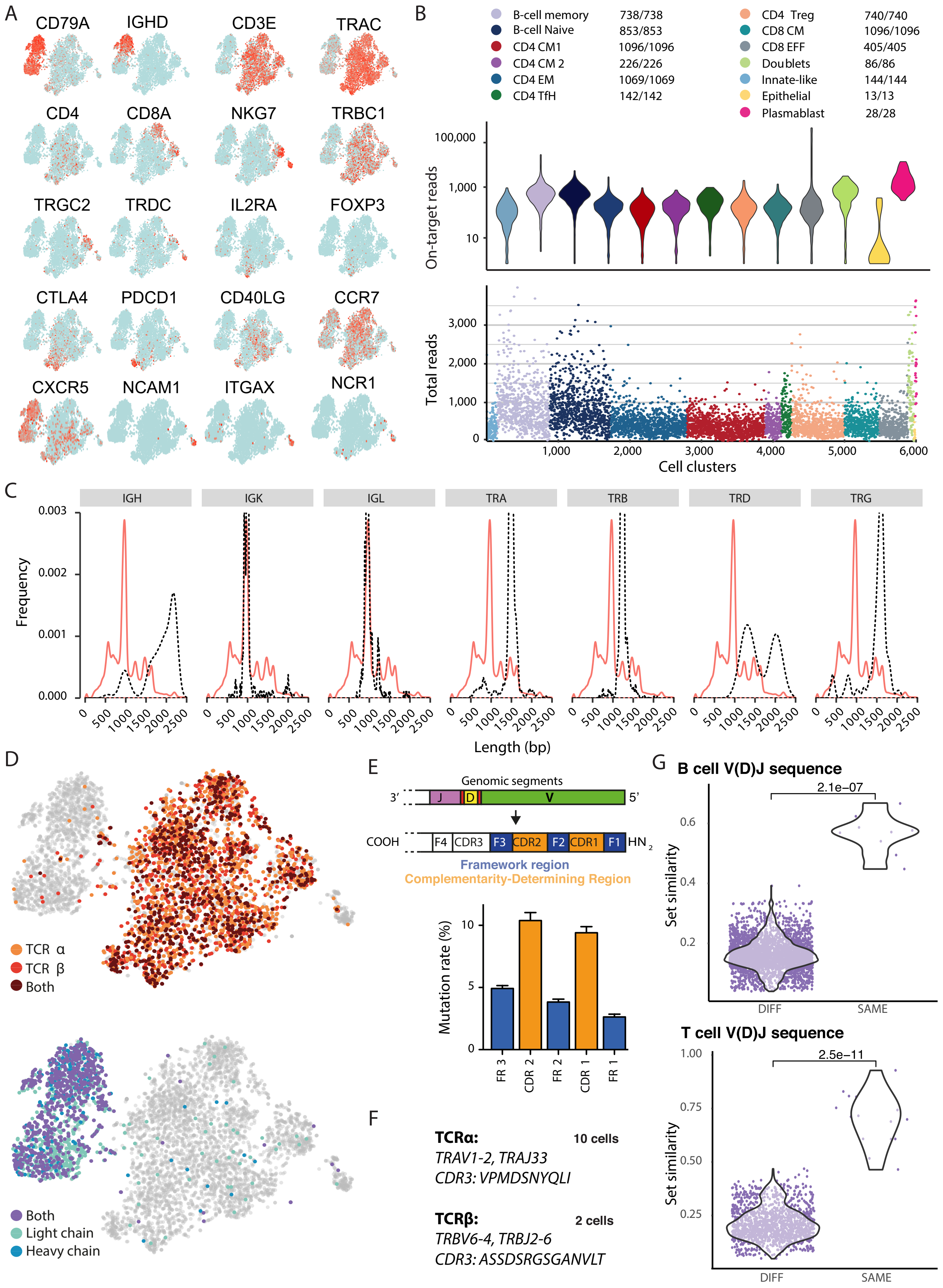

A
CD79A
IGHD
CD3E
TRAC
CD4
CD8A
NKG7
TRBC1
TRGC2
TRDC
IL2RA
FOXP3
CTLA4
PDCD1
CD40LG
CCR7
CXCR5
NCAM1
ITGAX
NCR1
B
B-cell memory 738/738
B-cell Naive 853/853
CD4 CM1 1096/1096
CD4 CM 2 226/226
CD4 EM 1069/1069
CD4 TfH 142/142
CD4 Treg 740/740
CD8 CM 1096/1096
CD8 EFF 405/405
Doublets 86/86
Innate-like 144/144
Epithelial 13/13
Plasmablast 28/28
On-target reads
Total reads
Cell clusters
C
IGH
IGK
IGL
TRA
TRB
TRD
TRG
Frequency
Length (bp)
D
TCR α
TCR β
Both
Both
Light chain
Heavy chain
E
Genomic segments
3′
J
D
V
5′
COOH
F4
CDR3
F3
CDR2
F2
CDR1
F1
HN 2
Framework region
Complementarity-Determining Region
Mutation rate (%)
FR 3
CDR 2
FR 2
CDR 1
FR 1
F
TCRα: 10 cells
TRAV1-2, TRAJ33
CDR3: VPMDSNYQLI
TCRβ: 2 cells
TRBV6-4, TRBJ2-6
CDR3: ASSDSRGSGANVLT
G
B cell V(D)J sequence
2.1e−07
Set similarity
DIFF
SAME
T cell V(D)J sequence
2.5e−11
Set similarity
DIFF
SAME

Supplementary Figure 5. Additional measurements of RAGE-seq on a human lymph node. **(a)** t-SNE analysis of key canonical gene expression markers used to identify cell type for each cluster. Briefly, CD4 CM 1: *CD4*+ *CD8A*- *CCR7*+, CD4 EM: *CD4*+ *CD8A*- *CCR7*-, B-cell naïve: *CD79A*+ *IGHD*+ *IGHG1*- *IGHGA*-, B-cell memory: *CD79A*+, *IGHD*- *IGHA1*+ *IGHG1*+, CD4 Treg: *CD4*+ *FOXP3*+ *IL2RA*+ *CTLA4*+, CD4 Tfh: *CD4*+ *CXCR5*+ *PDCD1*+ *CD40LG*+ *ILR2RA*- *FOXP3*-, Epithelial: *EPCAM*+ (not shown), Doublet: *CD3E*+ *CD79A*+ *TRAC*+ *IGHD*+ *IGHM*+ Plasmablast: *CD79A*+ (*JCHAIN*+ not shown), CD8 CM: *CD8A*+ *CD4*- *CCR7*+, CD8 EFF: *CD8A*+ *CD4*- *CCR7*- (*GZMA/B/K*+ *GNLY*+ not shown), CD4 CM 2: *CD4*+ *CD8*- *CCR7*+, Innate-like: *CD3E*+ *NKG7*+ *TRDC*+ *NCR1*+ *NCAM1*+. **(b)** The number of on-target nanopore reads for each cell population identified in the lymph node (top panel) and the recovery of cell barcodes for each cell within each cell population (bottom panel). The number of barcodes recovered is shown above the top panel. **(c)** The nucleotide length for assembled antigen receptor transcripts for each receptor chain identified across all cells in the lymph node. The overall nanopore sequencing read length distribution is shown as solid and the assembled contigs as dashed lines. **(d)** t-SNE plot of the assignment of TCR (top panel) and BCR (bottom panel) chains for each cell in the lymph node. **(e)** Mutation rate of the framework and complementarity regions of the heavy chain V regions that have been assigned to memory B cells. **(f)** TCRα and TCRβ chain sequence of T cells assigned MAIT-associated TCRs. **(g)** Jaccard set similarity score of top 250 raw UMI gene counts across all B cells (top) and T cells (bottom) with shared (SAME) V(D)J sequences and those with dissimilar (DIFF) V(D)J sequences within each respective cell type cluster. Only cells assigned paired TCR or paired BCR chains were analyzed. Significance was calculated via the corrected Wilcoxon test.

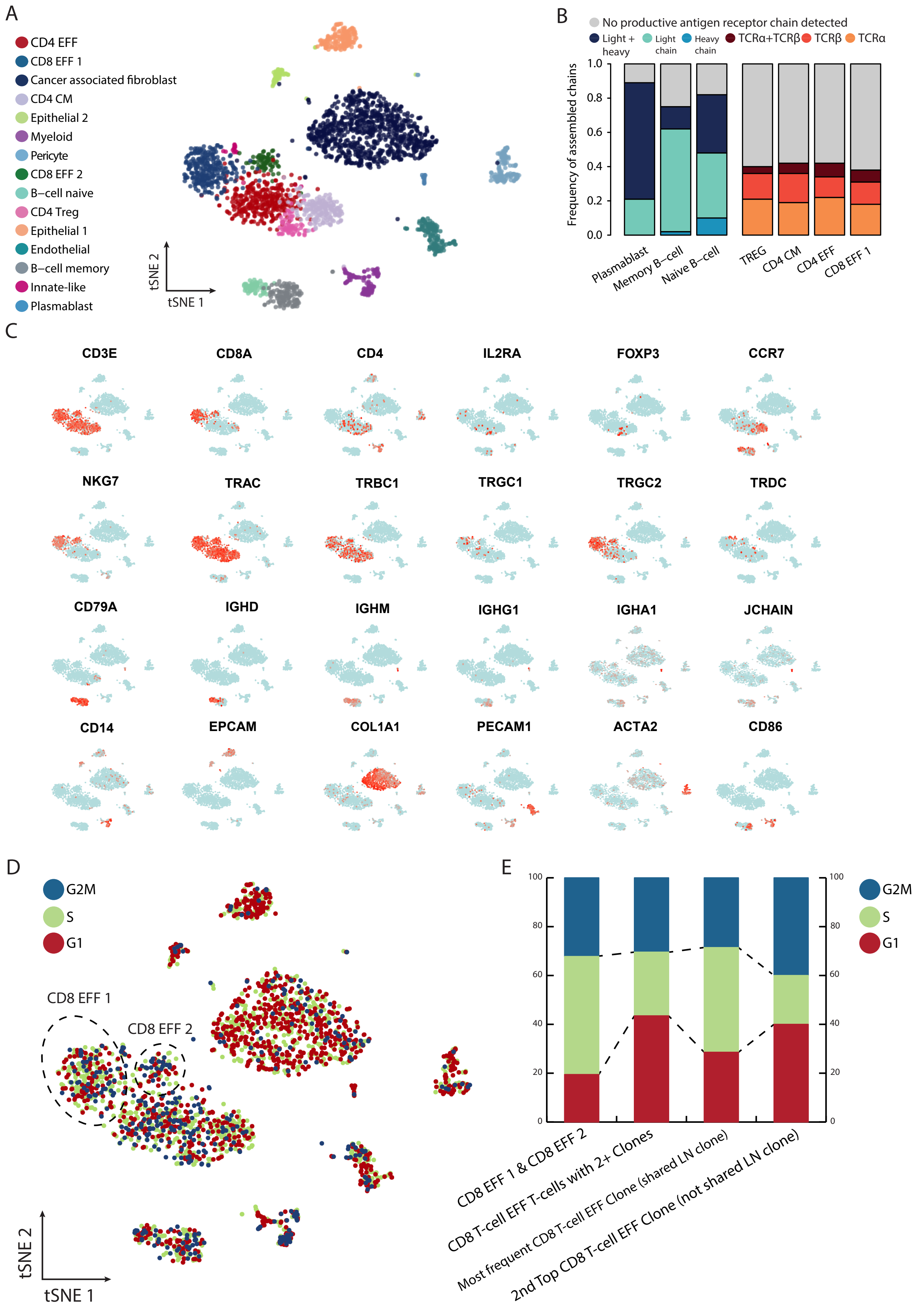

A
CD4 EFF
CD8 EFF 1
Cancer associated fibroblast
CD4 CM
Epithelial 2
Myeloid
Pericyte
CD8 EFF 2
B–cell naive
CD4 Treg
Epithelial 1
Endothelial
B–cell memory
Innate-like
Plasmablast
tSNE 2
tSNE 1
B
No productive antigen receptor chain detected
Light + heavy
Light chain
Heavy chain
TCRα+TCRβ
TCRβ
TCRα
Frequency of assembled chains
1.0
0.8
0.6
0.4
0.2
0.0
Plasmablast
Memory B–cell
Naive B–cell
TREG
CD4 CM
CD4 EFF
CD8 EFF 1
C
CD3E
CD8A
CD4
IL2RA
FOXP3
CCR7
NKG7
TRAC
TRBC1
TRGC1
TRGC2
TRDC
CD79A
IGHD
IGHM
IGHG1
IGHA1
JCHAIN
CD14
EPCAM
COL1A1
PECAM1
ACTA2
CD86
D
G2M
S
G1
CD8 EFF 1
CD8 EFF 2
tSNE 2
tSNE 1
E
100
80
60
40
20
0
G2M
S
G1
CD8 EFF 1 & CD8 EFF 2
CD8 T-cell EFF T-cells with 2+ Clones
Most frequent CD8 T-cell EFF Clone (shared LN clone)
2nd Top CD8 T-cell EFF Clone (not shared LN clone)

Supplementary Figure 6. Additional measurements of RAGE-seq on a tumour. **(a)** t-SNE analysis of 2,493 cells from a breast cancer tumor generated from short-read sequencing data. Number of cells: B-cell memory, 128; B-cell naïve, 68; cancer associated fibroblast, 773; CD4 CM; CD4 Treg, 67; CD4 CM, 323; CD4 EFF, 173; CD8 EFF 1, 257; CD8 EFF 2, 72; Endothelial, 144; Epithelial 1, 144; Epithelial 2, 57. Innate-like, 16; Myeloid, 113; Pericyte, 138; Plasmablast, 19. **(b)** Assignment of TCR chains to each T population and BCR chains to each B cell population identified in (a). **(c)** t-SNE plots of key canonical gene expression markers used to identify cell types in (a). Briefly, CD4 EFF: *CD4*+ *CD8*- *CCR7*-, CD8 EFF 1: *CD8*+ *CD4*- *CCR7*- *TRAC*+ (*GNLY*+ *GZMB*+, not shown), Cancer associated fibroblast: *COL1A1*+, CD4 CM: *CD4*+ *CD8*- *CCR7*+ , Epithelial 2: *EPCAM*+, Myeloid: *CD14*+ *CD86*+, Pericyte: *ACTA2*+, CD8 EFF 2: *CD8*+ *CD4*- *TRAC*+ *CCR7*- (*GZMA*+ *GZMK*+, not shown), B-cell Naïve: *CD79*+, *IGHD*+, *IGHG1*- *IGHA1*-, CD4 Treg: *CD4*+ *FOXP3*+ *IL2RA*+, Epithelial 1: *EPCAM*+, Endothelial 1: *PECAM1*, B-cell memory: *CD79A*+, *IGHD*- *IGHA1*+ *IGHG1*+, Innate-like: *CD3E*+ *TRDC*+, Plasmablast: *CD79A*+, *JCHAIN*+. **(d)** t-SNE anlaysis of cell cycle phase (G1, G2M or S) of all cells identified in the tumor. **(e)** Proportion of cell cycle phase of all cells identified within the tumor CD8 T-cell EFF clusters and the top two expanded CD8 T-cell EFF clones. Expanded clones were measured by the frequency of shared TCRβ V(D)J sequences. CD8 EFF 1 CD8 EFF 2 (n=329). T-cells with 2+ clones (n=23). Most frequent CD8 clone: V: *TRBV7-9* J: *TRBJ2-3*, CDR3: ASSLAGRVPGDTQY (n=7) and second top CD8 clone: *TRBV7-9 TRBJ2-2*, ASSLELTGELF (n=5).

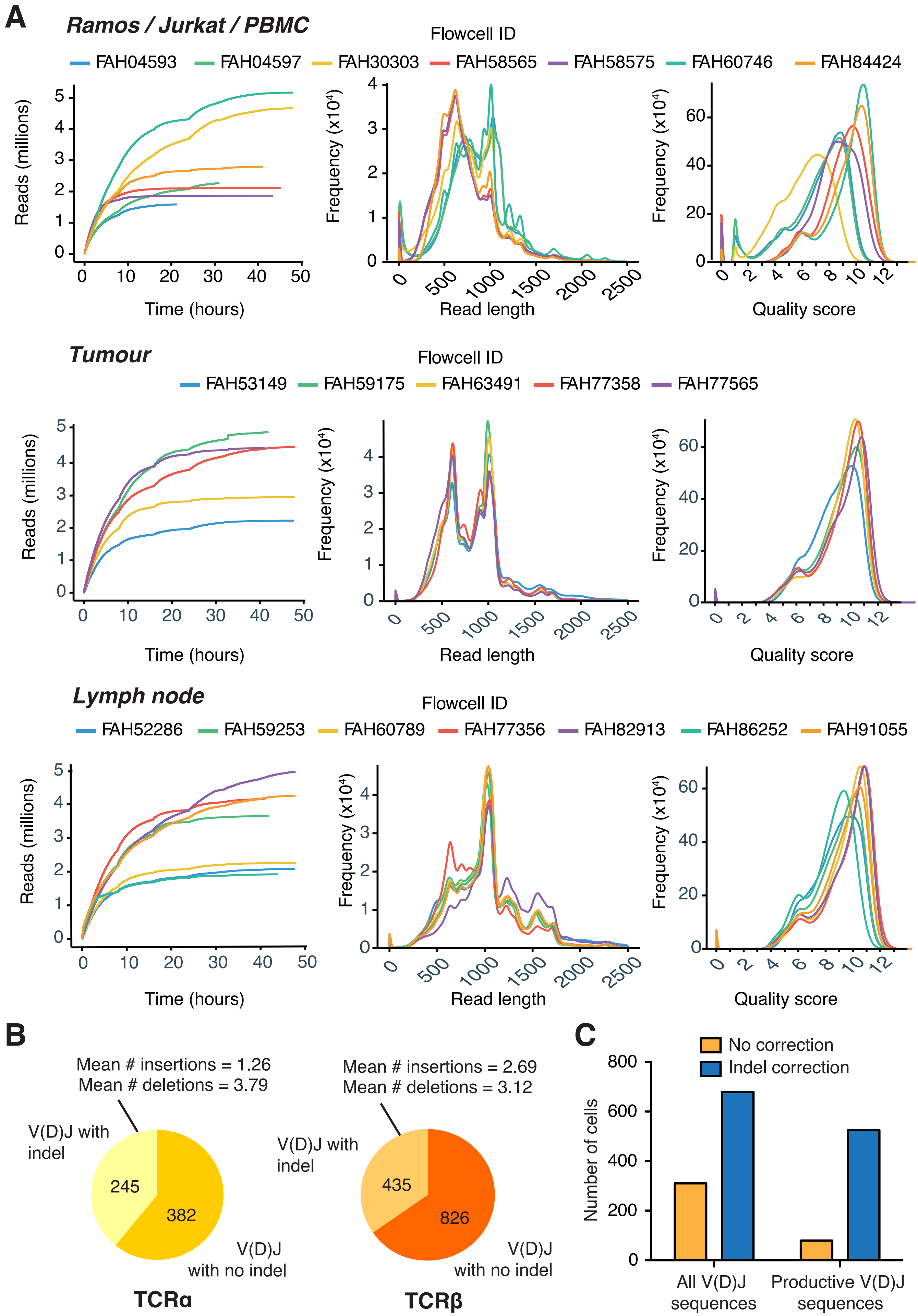

A
Ramos / Jurkat / PBMC
Flowcell ID
FAH04593 FAH04597 FAH30303 FAH58565 FAH58575 FAH60746 FAH84424
Reads (millions)
Time (hours)
Frequency (x10⁴)
Read length
Quality score
Tumour
FAH53149 FAH59175 FAH63491 FAH77358 FAH77565
Lymph node
FAH52286 FAH59253 FAH60789 FAH77356 FAH82913 FAH86252 FAH91055
B
Mean # insertions = 1.26
Mean # deletions = 3.79
V(D)J with indel
245
382
V(D)J with no indel
TCRα
Mean # insertions = 2.69
Mean # deletions = 3.12
435
826
TCRβ
C
No correction
Indel correction
Number of cells
All V(D)J sequences
Productive V(D)J sequences

Supplementary Figure 7. Nanopore sequencing statistics and indel correction. **(a)** Number of reads, read length and quality score for cell line, tumour and lymph node samples. Color denotes each flowcell the run was performed on (R9.4 or R9.5 chemistry). **(b)** The total number of TCR chains assigned to Jurkat cells (n=1463) that carry indels in their *TRAV* or *TRBV* gene before indel correction (see Methods). TCR chains include those with non-productive CDR3 sequences. **(c)** The effect of indel correction on antigen receptor chain recovery. Shown are all productive BCR and TCR chains recovered across the cell line experiment (all Jurkat, Ramos and monocyte cells). 'All V(D)J sequences' refers to both productive and non-productive antigen receptor sequences.

Supplementary Table 1. Number of on-target nanopore reads post sequence capture.

	Total read count	On-target	Demultiplexed reads	Demux. On-target
Cell lines	20,346,396	8,715,631(42.8%)	3,805,076(18.7%)	1,915,352(50.3%)
Lymph Node	23,044,761	12,560,425(54.5%)	3,380,621(14.7%)	1,989,680(58.9%)
Tumour	16,601,436	8,611,279(51.9%)	3,069,468(18.5%)	1,899,447(61.9%)

Supplementary Table 2. Percentage of Illumina reads assigned to the final cell barcode lists.

	Ramos	Lymph Node	Tumor
Total Illumina reads	690,512,282	309,532,978	107,790,387
Final cell barcodes list	532,968,514	171,613,482	65,527,299
Percentage of total	77.2	55.4	60.8

Supplementary Table 3. Comparison of RAGE-Seq against SmartSeq2.

	#Jurkat cells	TRA recovery	TRB Recovery	Paired	Cost/Cell (AUD)
RAGE-Seq	1463	472	856	277	$3.70
SmartSeq + VDJ-Puzzle	28	22	25	21	$90.92

Supplementary Table 4. Cost-breakdown of RAGE-Seq and Smart-Seq2.[1]

Items	Supplier	Cost per experiment (AUD)	Cost per cell (AUD)
		RAGE-seq	
Capture probes (NimbleGen)	Roche	$1173	$0.31
SeqCap EZ accessory kit	Roche	$46	$0.013
Hybridisation and wash kit	Roche	$19.8	$0.0053
KAPA Hotstart HiFi ReadyMix	Kapa Biosystems	$12.2	$0.0031
Dynabeads M-270 Streptavidin	ThermoFisher	$65.53	$0.018
AMPure XP magnetic beads	Agencourt	$20.5	$0.0055
10X Chromium capture and library preparation	10X Genomics	$2776.50	$0.74
MinION library prep and sequencing (x7)	Oxford Nanopore	$6125	$1.64
NextSeq 500/550 Mid Output v2 kit (150 cycles)	Illumina	$1339.50	$0.36
NextSeq 500/550 High Output v2 kit (150 cycles)	Illumina	$3522	$0.94
		Smart-Seq2	
Recombinant RNase Inhibitor	Clontech		$2.04
SuperScript II Reverse Transcriptase	Invitrogen		$3.48
KAPA Hotstart HiFi ReadyMix	Kapa Biosystems		$1.22
Nextera XT Index Kit	Illumina		$3.15
Nextera XT DNA Library Prep Kit	Illumina		$39.0
AMPure XP magnetic beads	Agencourt		$1.75
LabChip GX Touch 24	PerkinElmer		$1.50
PicoGreen	ThermoFisher		$0.82
dNTP Mix	ThermoFisher		$0.14
Oligonucleotides	Exiqon		$3.40
NextSeq 500/550 High Output v2 kit (300 cycles) $\sim 2.5 \times 10^6$ reads/cell	Illumina		$35.28

[1] RAGE-Seq had 3,743 cells in the final dataset. Costs were calculated for the cell line experiment for RAGE-Seq and for 28 Jurkat cells for Smart-Seq2. The cost per experiment for Smart-Seq2 cannot be calculated in the same way as RAGE-Seq because the user can choose the exact number of cells to be sequenced using Smart-Seq2.

Supplementary Table 5. Assignment of TCRα and TCRβ chains to cells assigned TCRγ and/or TCRδ chains.

Assigned chain (number of cells)	TCRα	TCRβ	TCR$\alpha\beta$
TCRγ only (14)	1	1	0
TCRδ only (84)	16	12	5
TCRγ + TCRδ (11)	0	0	0

Supplementary Table 6. Comparison to other droplet-based scRNA-Seq platforms[2]

	RAGE-Seq	10X 5' V(D)J	DART-Seq	ScISOr-Seq
Description	Transcriptome +TCR+BCR	Transcriptome +TCR/BCR	Transcriptome + BCR	Transcriptome only
Throughput	$> 10^4$	$> 10^4$	$> 10^4$	$> 10^4$
Estimated cost/cell (AUD)	$2.50	$1.60	N/A	$6.30
Full-length mRNA	Yes	No	NA	Yes
Reports antigen receptors	Yes	Yes	Yes	No
Paired TCR recovery (%)	16.9	55.1	N/A	N/A
Paired BCR recovery (%)	42.6	59.1	20.1	N/A
Reports TCR$\gamma\delta$	Yes	No	No	No
Reports BCR somatic hypermutation	Yes	No	No	N/A
Reports if BCR is secreted or membrane-bound	Yes	No	No	N/A

[2] 10X V(D)J data was obtained from: `https://support.10xgenomics.com/single-cell-vdj/datasets` (TCR and Ig enrichment from PBMCs of a healthy donor). Costs are estimated for sequencing 5,000 T or B cells using a single 10X capture. Sequencing costs are estimated using the NextSeq platform. The lymph node sample was used to calculate the costs for RAGE-Seq. Cost per cell is lower than reported in Supplementary Table 4 for RAGE-Seq because more cells were sequenced for the lymph node sample than the cell line sample.

Supplementary Table 7. Targeted gene segments.

IGHA1	IGHA2	IGHD	IGHE	IGHG1	IGHG2
IGHG3	IGHG4	IGHJ1	IGHJ2	IGHJ3	IGHJ4
IGHJ5	IGHJ6	IGHM	IGHV1-18	IGHV1-2	IGHV1-24
IGHV1-3	IGHV1-45	IGHV1-46	IGHV1-58	IGHV1-69	IGHV1-69-2
IGHV2-26	IGHV2-5	IGHV2-70	IGHV3-11	IGHV3-13	IGHV3-15
IGHV3-16	IGHV3-20	IGHV3-21	IGHV3-23	IGHV3-30	IGHV3-33
IGHV3-35	IGHV3-38	IGHV3-43	IGHV3-48	IGHV3-49	IGHV3-53
IGHV3-64	IGHV3-66	IGHV3-7	IGHV3-72	IGHV3-73	IGHV3-74
IGHV4-28	IGHV4-31	IGHV4-34G17	IGHV4-39	IGHV4-4	IGHV4-59
IGHV4-61	IGHV5-51	IGHV6-1	IGHV7-81	IGKC	IGKJ1
IGKJ2	IGKJ3	IGKJ4	IGKJ5	IGKV1-12	IGKV1-16
IGKV1-17	IGKV1-27	IGKV1-33	IGKV1-37	IGKV1-39	IGKV1-5
IGKV1-6	IGKV1-8	IGKV1-9	IGKV1D-12	IGKV1D-13	IGKV1D-16
IGKV1D-17	IGKV1D-33	IGKV1D-37	IGKV1D-39	IGKV1D-42	IGKV1D-43
IGKV1D-8	IGKV2-24	IGKV2-28	IGKV2-30	IGKV2-40	IGKV2D-24
IGKV2D-26	IGKV2D-28	IGKV2D-29	IGKV2D-30	IGKV2D-40	IGKV3-11
IGKV3-15	IGKV3-20	IGKV3-7	IGKV3D-11	IGKV3D-15	IGKV3D-20
IGKV3D-7	IGKV4-1	IGKV5-2	IGKV6-21	IGKV6D-21	IGKV6D-41
IGLC1	IGLC2	IGLC3	IGLC7	IGLJ1	IGLJ2
IGLJ3	IGLJ4	IGLJ5	IGLJ6	IGLJ7	IGLV1-36
IGLV1-40	IGLV1-44	IGLV1-47	IGLV1-50	IGLV1-51	IGLV10-54
IGLV11-55	IGLV2-11	IGLV2-14	IGLV2-18	IGLV2-23	IGLV2-33
IGLV2-8	IGLV3-1	IGLV3-10	IGLV3-12	IGLV3-16	IGLV3-19
IGLV3-21	IGLV3-22	IGLV3-25	IGLV3-27	IGLV3-32	IGLV3-9
IGLV4-3	IGLV4-60	IGLV4-69	IGLV5-37	IGLV5-45	IGLV5-48
IGLV5-52	IGLV6-57	IGLV7-43	IGLV7-46	IGLV8-61	IGLV9-49
TRAC	TRAJ1	TRAJ10	TRAJ11	TRAJ12	TRAJ13
TRAJ14	TRAJ16	TRAJ17	TRAJ18	TRAJ19	TRAJ2
TRAJ20	TRAJ21	TRAJ22	TRAJ23	TRAJ24	TRAJ25
TRAJ26	TRAJ27	TRAJ28	TRAJ29	TRAJ3	TRAJ30
TRAJ31	TRAJ32	TRAJ33	TRAJ34	TRAJ35	TRAJ36
TRAJ37	TRAJ38	TRAJ39	TRAJ4	TRAJ40	TRAJ41
TRAJ42	TRAJ43	TRAJ44	TRAJ45	TRAJ46	TRAJ47
TRAJ48	TRAJ49	TRAJ5	TRAJ50	TRAJ52	TRAJ53
TRAJ54	TRAJ56	TRAJ57	TRAJ58	TRAJ59	TRAJ6
TRAJ61	TRAJ7	TRAJ9	TRAV1-1	TRAV1-2	TRAV10
TRAV12-1	TRAV12-2	TRAV12-3	TRAV13-1	TRAV13-2	TRAV14DV4
TRAV16	TRAV17	TRAV18	TRAV19	TRAV2	TRAV20
TRAV21	TRAV22	TRAV23DV6	TRAV24	TRAV25	TRAV26-1
TRAV26-2	TRAV27	TRAV29DV5	TRAV3	TRAV30	TRAV34
TRAV36DV7	TRAV38-1	TRAV38-2DV	TRAV39	TRAV4	TRAV40
TRAV41	TRAV5	TRAV6	TRAV7	TRAV8-1	TRAV8-2
TRAV8-3	TRAV8-4	TRAV8-6	TRAV8-7	TRAV9-1	TRAV9-2
TRBC1	TRBC2	TRBJ1-1	TRBJ1-2	TRBJ1-3	TRBJ1-4
TRBJ1-5	TRBJ1-6	TRBJ2-1	TRBJ2-2	TRBJ2-3	TRBJ2-4
TRBJ2-5	TRBJ2-6	TRBJ2-7	TRBV10-1	TRBV10-2	TRBV10-3
TRBV11-1	TRBV11-2	TRBV11-3	TRBV12-3	TRBV12-4	TRBV12-5
TRBV13	TRBV14	TRBV15	TRBV16	TRBV17	TRBV18
TRBV19	TRBV2	TRBV20-1	TRBV23-1	TRBV24-1	TRBV25-1
TRBV27	TRBV28	TRBV29-1	TRBV3-1	TRBV30	TRBV4-1
TRBV4-2	TRBV5-1	TRBV5-3	TRBV5-4	TRBV5-5	TRBV5-6
TRBV5-7	TRBV6-1	TRBV6-2	TRBV6-4	TRBV6-5	TRBV6-6
TRBV6-7	TRBV6-8	TRBV7-1	TRBV7-2	TRBV7-3	TRBV7-4
TRBV7-6	TRBV7-7	TRBV7-9	TRBV9	TRDC	TRDD2
TRDJ1	TRDJ2	TRDJ3	TRDJ4	TRDV1	TRDV2
TRDV3	TRGC1	TRGC2	TRGJ1	TRGJ2	TRGJP
TRGJP1	TRGJP2	TRGV10	TRGV11	TRGV2	TRGV3
TRGV4	TRGV5	TRGV8	TRGV9		

Supplementary Table 8. List of samples, chemistries, flowcell identification numbers, and manufacturer software versions.

Sample	Flowcell ID	Flowcell Chemistry	Kit	Albacore
Tumour	FAH59175	FLO-MIN106	SQK-LSK108	2.2.7
	FAH63491			
	FAH77585			
	FAH77358			
	FAH53149			2.1.3
	FAH53149			
	FAH89946			N/A
Lymph Node	FAH59253	FLO-MIN106	SQK-LSK108	2.2.7
	FAH60789			
	FAH77356			
	FAH82913			
	FAH52286			2.1.3
	FAH52286			
	FAH86252			N/A
	FAH82560			
Cell lines	FAH58575	FLO-MIN106	SQK-LSK108	2.2.7
	FAH58565			
	FAH60746			
	FAH84424			
	FAH04597	FLO-MIN107	SQK-LSK308 (as 1D)	2.1.3
	FAH04593		SQK-LSK108	
	FAH30303			
