# Supplementary material for: High-throughput targeted long-read single cell sequencing reveals the clonal and transcriptional landscape of lymphocytes
Source: Nat Commun. 2019 Jul 16;10:3120. doi: 10.1038/s41467-019-11049-4 (PMC6635368; doi:10.1038/s41467-019-11049-4)
Supplement: Supplementary file 3 — Description of Additional Supplementary Files [file 41467_2019_11049_MOESM3_ESM.pdf]

Description of Additional Supplementary Files

File Name: Supplementary Data 1
Description: Assignment of unique V(D)J chains to lymph node and tumor.
