# Supplementary material for: High-throughput targeted long-read single cell sequencing reveals the clonal and transcriptional landscape of lymphocytes
Source: Nat Commun. 2019 Jul 16;10:3120. doi: 10.1038/s41467-019-11049-4 (PMC6635368; doi:10.1038/s41467-019-11049-4)
Supplement: Supplementary file 5 — Reporting Summary [file 41467_2019_11049_MOESM5_ESM.pdf]

nature research

Corresponding author(s): Swarbrick, Smith, Goodnow

Last updated by author(s): Jun 6, 2019

Reporting Summary

Nature Research wishes to improve the reproducibility of the work that we publish. This form provides structure for consistency and transparency in reporting. For further information on Nature Research policies, see Authors & Referees and the Editorial Policy Checklist.

Statistics

For all statistical analyses, confirm that the following items are present in the figure legend, table legend, main text, or Methods section.

n/a	Confirmed	
☐	☒	The exact sample size (*n*) for each experimental group/condition, given as a discrete number and unit of measurement
☐	☒	A statement on whether measurements were taken from distinct samples or whether the same sample was measured repeatedly
☐	☒	The statistical test(s) used AND whether they are one- or two-sided<br>*Only common tests should be described solely by name; describe more complex techniques in the Methods section.*
☐	☒	A description of all covariates tested
☐	☒	A description of any assumptions or corrections, such as tests of normality and adjustment for multiple comparisons
☐	☒	A full description of the statistical parameters including central tendency (e.g. means) or other basic estimates (e.g. regression coefficient) AND variation (e.g. standard deviation) or associated estimates of uncertainty (e.g. confidence intervals)
☐	☒	For null hypothesis testing, the test statistic (e.g. *F*, *t*, *r*) with confidence intervals, effect sizes, degrees of freedom and *P* value noted<br>*Give P values as exact values whenever suitable.*
☒	☐	For Bayesian analysis, information on the choice of priors and Markov chain Monte Carlo settings
☐	☒	For hierarchical and complex designs, identification of the appropriate level for tests and full reporting of outcomes
☐	☒	Estimates of effect sizes (e.g. Cohen's *d*, Pearson's *r*), indicating how they were calculated

Our web collection on statistics for biologists contains articles on many of the points above.

Software and code

Policy information about availability of computer code

Data collection	Data generated in this study have been deposited in deposited in European Nucleotide Archive under the primary accession code PRJEB28878.
Data analysis	All code used for the computational pipeline of this manuscript can be found at (https://github.com/KCCG/rageseq).

For manuscripts utilizing custom algorithms or software that are central to the research but not yet described in published literature, software must be made available to editors/reviewers. We strongly encourage code deposition in a community repository (e.g. GitHub). See the Nature Research guidelines for submitting code & software for further information.

Data

Policy information about availability of data

All manuscripts must include a data availability statement. This statement should provide the following information, where applicable:

- Accession codes, unique identifiers, or web links for publicly available datasets
- A list of figures that have associated raw data
- A description of any restrictions on data availability

Data generated in this study have been deposited in deposited in European Nucleotide Archive under the primary accession code PRJEB28878.

Field-specific reporting

Please select the one below that is the best fit for your research. If you are not sure, read the appropriate sections before making your selection.

☒ Life sciences ☐ Behavioural & social sciences ☐ Ecological, evolutionary & environmental sciences

October 2018

Life sciences study design

All studies must disclose on these points even when the disclosure is negative.

Sample size	No statistical tests were chosen. These are exploratory studies, thus single models and patients were studied
Data exclusions	No data were excluded
Replication	All attempts at replication were successful
Randomization	This is an observational study and there are no experimental perturbations
Blinding	Blinding was not relevant as our analysis does not include quantitative measurements of differences between experimental groups

Reporting for specific materials, systems and methods

We require information from authors about some types of materials, experimental systems and methods used in many studies. Here, indicate whether each material, system or method listed is relevant to your study. If you are not sure if a list item applies to your research, read the appropriate section before selecting a response.

Materials & experimental systems

n/a	Involved in the study	
☒	☐	Antibodies
☐	☒	Eukaryotic cell lines
☒	☐	Palaeontology
☒	☐	Animals and other organisms
☐	☒	Human research participants
☒	☐	Clinical data

Methods

n/a	Involved in the study	
☒	☐	ChIP-seq
☒	☐	Flow cytometry
☒	☐	MRI-based neuroimaging

Eukaryotic cell lines

Policy information about cell lines

Cell line source(s)	Ramos cell line: ATCC. Jurkat cell line: University of California San Fransisco
Authentication	Both the Ramos cell line and Jurkat cell line where authenticated by PCR amplification and Sanger sequencing of their antigen receptors (BCR or TCR) and these sequences compared to the published sequences.
Mycoplasma contamination	The cell lines were not tested for mycoplasma contamination.
Commonly misidentified lines (See ICLAC register)	*Name any commonly misidentified cell lines used in the study and provide a rationale for their use.*

Human research participants

Policy information about studies involving human research participants

Population characteristics	A single human subject was used. Their characteristics are described in the manuscript
Recruitment	The subject was recruited at the time of surgical consulation as part of a routine research consenting process.
Ethics oversight	This study is approved by the St Vincents and Mater Health Human Research Ethics Committee

Note that full information on the approval of the study protocol must also be provided in the manuscript.
